# The Special K Constellation, a Rare Presentation of Ketamine Use: A Case Report

**DOI:** 10.7759/cureus.4766

**Published:** 2019-05-28

**Authors:** Jarrett Williams, Edmund Hsu, Adam Flamer-Caldera, Ye Jung Ferrabolli

**Affiliations:** 1 Emergency Medicine, Mount Sinai St. Luke's West, New York, USA; 2 Emergency Medicine, Mount Sinai St. Luke's - Roosevelt Hospital Center, New York, USA; 3 Emergency Medicine, Mount Sinai St Luke's, New York, USA

**Keywords:** ketamine, pneumorrhachis

## Abstract

Ketamine is commonly used in the emergency medicine setting, but also as a recreational drug. There have been many animal studies investigating ketamine, but little data on long-term clinical use of ketamine in humans. In this case presentation, a 22-year-old international male student presented with crepitus and cachexia and was found to have extensive subcutaneous emphysema, pneumorrhachis/intraspinal air, pneumomediastinum, and multiorgan failure. In this case report, we discuss how ketamine abuse is the likely cause of these findings.

## Introduction

Ketamine was developed in 1962 and approved in 1970 by the federal government for human use as a battlefield anesthetic agent. Today, ketamine is commonly not only used in the emergency medicine setting of intubation and procedural sedation, but also has gained wide recognition as a recreational drug where users can develop hallucinations, floating sensations, and dissociation [1, 2]. Ketamine, also known as 2-chlorophenyl-2-methylamino-cyclohexanone, is structurally related to phencyclidine [3]. It is a non-competitive antagonist of the N-methyl-D-aspartate (NMDA) receptor [4]. The antagonistic action on NMDA receptors may account for the dissociative effects of ketamine [2]. These effects are a result of reduced activation in the thalamocortical structures and increased activity in the limbic system and hippocampus.

In 2012, the United States reported the annual prevalence rate for ketamine in 12th graders as 1.5% [5]. Internationally, it was reported that 7.6% of registered drug users in China were using ketamine and the drug abuse registry for Hong Kong found that ketamine was the most popular psychotropic substance used in 2012 [6]. Twenty-nine percent of drug users in Hong Kong reported using ketamine. Sixty-one percent of those using ketamine were less than 21 years old [7, 8].

There have been many animal studies investigating ketamine, but there are little data on long-term clinical use of ketamine in humans. This case will examine an interesting clinical presentation and review briefly the current literature on ketamine toxicity.

## Case presentation

Initial presentation

**Figure 1:**
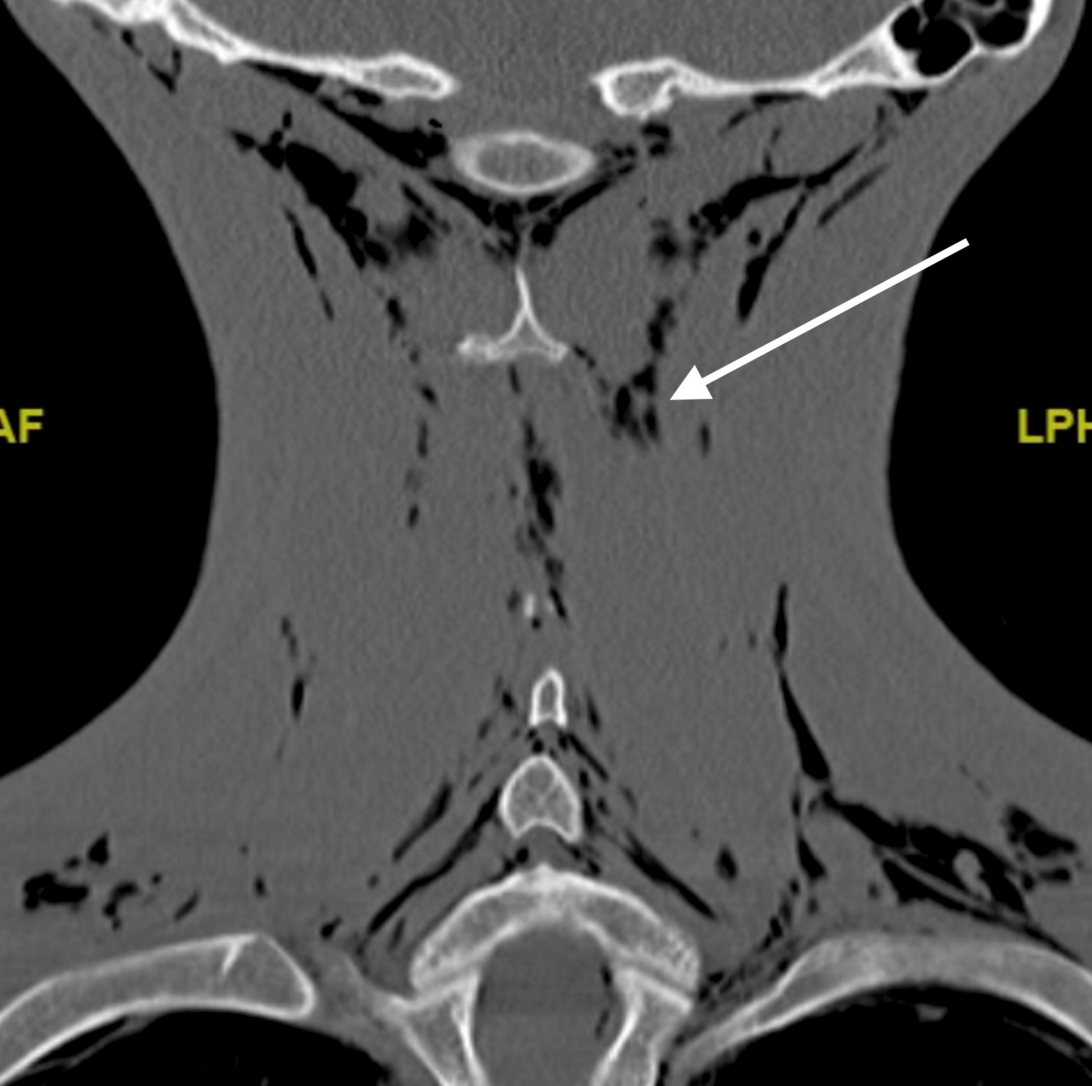
Coronal slice of cervical CT highlighting subcutaneous emphysema.

**Figure 2:**
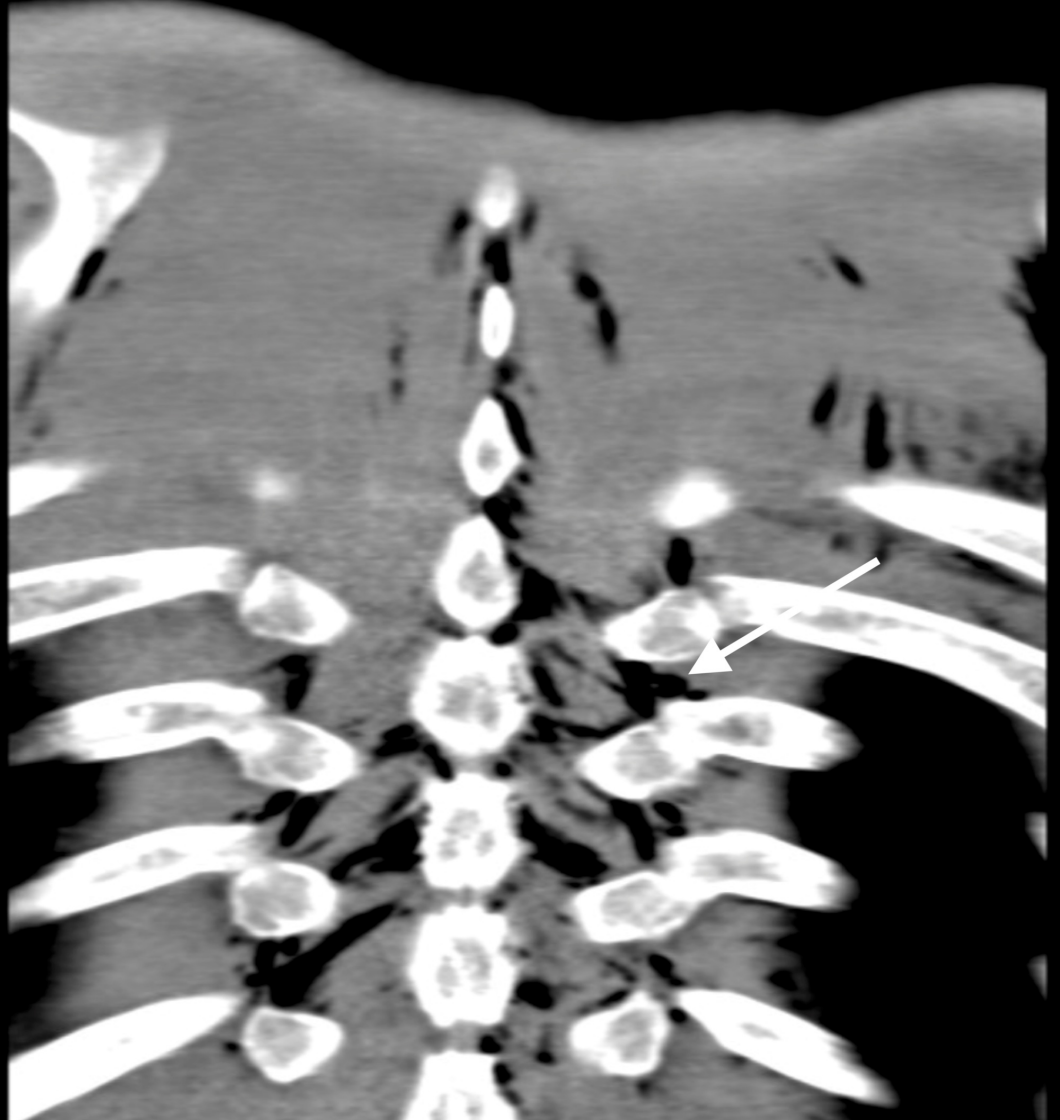
Coronal slice of chest CT highlighting pneumorrhachis and subcutaneous emphysema.

**Figure 3:**
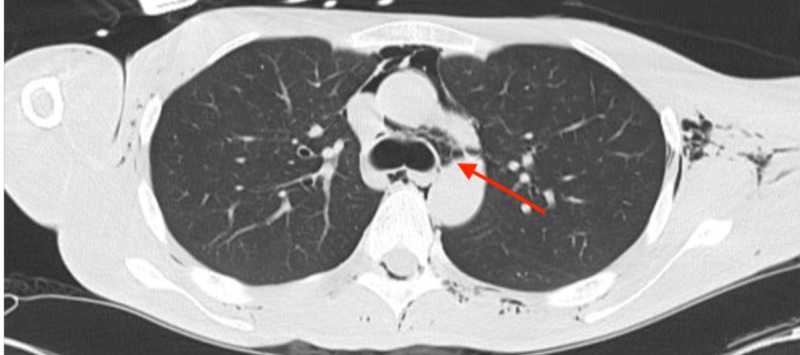
Axial slice of cervical CT highlighting pneumomediastinum, pneumorrhachis and further subcutaneous emphysema.

**Figure 4:**
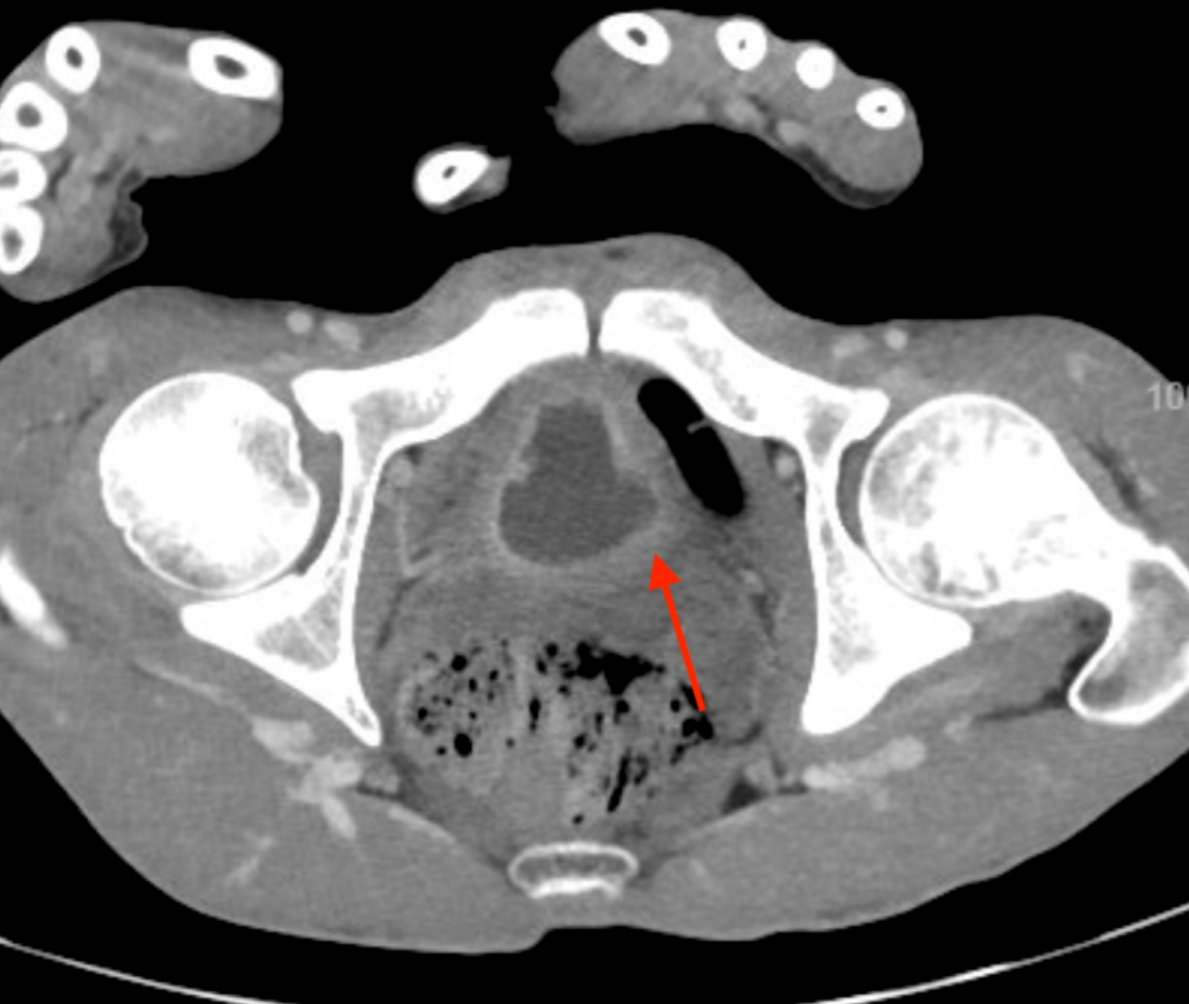
Mucosal enhancement of bladder wall.

**Figure 5:**
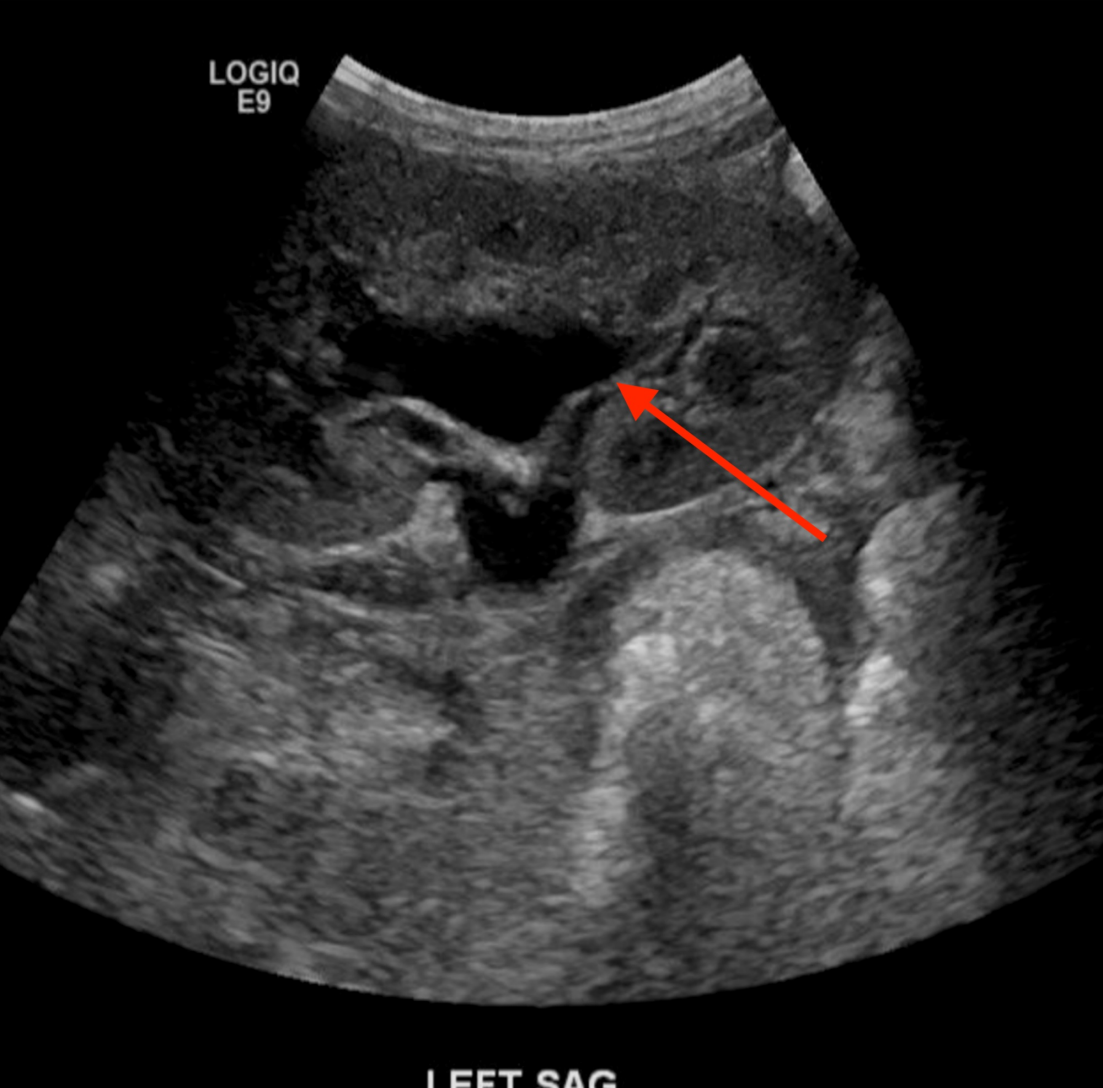
Renal hydronephrosis.

A 22-year-old international male student presented to a New York City (NYC) urban academic emergency department after being observed stumbling on the street outside the hospital. In triage, his initial vitals were notable for a blood pressure (BP) of 63/39 mmHg and a heart rate (HR) of 111 beats per minute (bpm). On physical exam, he was alert and oriented to person, place, and time, but visibly weak and noted to have severe cachexia upon removal of his clothing. His rectal temperature was 93.9 Fahrenheit (F). His only medical complaint was two months of abdominal pain and weight loss in excess of 30 lbs. He denied any other symptoms including fever, chills, nausea, vomiting, diarrhea, hematochezia, black colored stool, drug use, recent trauma or surgical procedures. Further physical examination was notable for crepitus over his left anterior neck and a flat, but diffusely tender abdomen. Bedside point of care ultrasound (POCUS) illustrated a collapsible inferior vena cava (IVC), hyper dynamic heart and good lung sliding bilaterally. Intravenous (IV) access was attained, and fluids and morphine were given for hypotension and pain, respectively. Initial point of care venous blood gas showed pH of 7.08, carbon dioxide (pCO2) of 39 mmHg, bicarbonate (HCO3) of 11.6 meq/L, sodium (Na) of 106 mmol/L, potassium (K) of 6.6 mmol/L, glucose of 117 mg/dL, and lactate of 4.7 mmol/L. His electrocardiogram (EKG) demonstrated peaked T waves. In the setting of hyperkalemia and consistent EKG changes, hyperkalemia protocol - Dextrose 50% (D50) fluids, insulin, albuterol, and calcium gluconate were given. Additionally, the patient was given vancomycin, cefepime, flagyl, IV fluids, and externally rewarmed. A portable chest X-ray showed diffuse subcutaneous emphysema and pneumomediastinum. Additional labs were notable for a white blood cell count (WBC) of 29.2, 94% neutrophils, absolute neutrophil count (ANC) of 29.6 k/ul. His alkaline phosphate was elevated to 1,198 with only mildly elevated liver enzymes (aspartate aminotransferase [AST], alanine aminotransferase [ALT] at 138U/L and 86 U/L, respectively). CT head, neck, chest and abdomen were performed and demonstrated extensive subcutaneous emphysema, pneumorrhachis/intraspinal air (Figures 1, 2), pneumomediastinum (Figure 3), liver biliary duct dilation, mucosal enhancement of entire bladder (Figure 4), and previously known hydronephrosis (Figure 5). Upon previous chart review, our patient had presented three months prior endorsing epigastric abdominal pain for one month that he was self-treating with intra-nasal ketamine three times a week. Upon further questioning however, he continued to deny drug use. Given the constellation of symptoms, multiorgan failure and CT findings, the patient was admitted to intensive care unit (ICU) for further workup.

Hospital course

The patient was placed on a non-rebreather in the ICU with resolution of palpable subcutaneous crepitus, pneumomediastinum, and pneumorrhachis. He underwent esophagram and abdominal CT, which showed no evidence of contrast extravasation. Abdominal ultrasound showed persistent intrahepatic biliary dilation and hypoechoic lesion in the right liver lobe. Renally, hydroureter and pelvis with slight hydronephrosis of the right kidney were present. Magnetic resonance cholangiopancreatography (MRCP) showed a pseudostricture with mild uniform intrahepatic biliary dilatation. The patient was transferred to floors from ICU after four days and gross hematuria continued, electrolytes normalized, leukocytosis resolved, and mental status improved. The patient was discharged from the hospital with follow-up with neurosurgery for pneumorrhachis, pulmonary for pneumothorax, urology for hematuria, and hepatology for elevated liver enzymes, predominantly cholestatic with preserved liver function with MRCP showing an isolated narrowing on hepatic duct and mild intrahepatic duct dilation.

## Discussion

In summary, this case describes a patient who presented with crepitus and cachexia and was found to have extensive subcutaneous emphysema, pneumorrhachis/intraspinal air, pneumomediastinum, and multiorgan failure, most likely due to ketamine abuse. There have been many animal studies investigating ketamine, but there are little data on long-term clinical use of ketamine in humans. There are multiple documented complications of ketamine toxicity, three of which our patient experienced.

Urinary tract abnormalities are the most commonly reported chronic toxic effect related to ketamine abuse. With chronic use, the drug injures the urinary bladder, causing ulcers, cystitis, and fibrosis. This can lead to urinary incontinence, hematuria, bladder overactivity and shrinkage, and, in the later stages, hydroureter and hydronephrosis [9]. The term “ketamine bladder syndrome” has been coined to describe this clinical entity. The smooth muscle relaxing property of ketamine was thought to be a pathogenic mechanism of urinary tract disease.

Ketamine can cause severe abdominal pain after daily high doses, known as “K pains” that are similar to severe gas pains [1]. The reported prevalence of upper gastrointestinal symptoms is up to 75%. Gastritis has been demonstrated in 85% of those who had endoscopy [10]. Complete relief of the symptoms is observed in most cases when patients abstain from the drug. With chronic or intermittent use, however, unusual biliary and hepatic complications have been described [11]. In a manner similar to its effects on the urinary tract, ketamine can also cause abnormalities in the biliary system with dilation and irregularity of the intra- and extra-hepatic bile ducts. Patients typically developed right upper quadrant pain and tenderness associated with elevations in serum alkaline phosphatase and aminotransferase levels, with minimal or no increase in bilirubin [11-13]. Biliary imaging may reveal dilation and irregularity of the intra- and extra-hepatic bile ducts with fusiform dilation of the common bile duct suggestive of choledochal cysts. Liver biopsy demonstrates changes suggestive of chronic liver obstruction or sclerosing cholangitis [14]. Discontinuation of ketamine is usually followed by slow improvement and the abnormalities found on biliary imaging may no longer be demonstrable several months later.

Lastly, frequent snorting/sniffing of ketamine can cause significant barotrauma. Passive apnea and/or cough that occur after sniffing can cause intra-alveolar hyper-pressure, which is responsible for alveolar rupture and air diffusion. Barotrauma is generated by increased intrapulmonary pressure and a subsequent high transmural gradient between the alveoli and the surrounding interstitial space. Allen et al. and Weissberg highlighted the proposed pathology associated with pneumomediastinum and pneumorrhachis. After the rupture, air diffuses to interstitial space, and then permeates the mediastinal soft tissue layers. The mediastinum communicates easily with deep cervical tissue layers and subcutaneous cervical space. Finally, mediastinal air migrates through the inter-vertebral foramens towards epidural space and the pneumorrhachis can form [15, 16]. Pneumorrhachis in particular is exceedingly rare and we could only find one prior report in the setting of ketamine use- a Hong Kong based journal, where the patients presented status post loss of consciousness (LOC) and found to have an elevated alkaline phosphatase (ALP), air in their chest and thoracic spine without pneumothorax or traumatic entry wound [17].

## Conclusions

Upon review of the literature, there are limited case reports of ketamine toxicity in the United States. In the existing reports, it appears patients have presented with individual complications of ketamine abuse but none, found on our brief literature review, with the constellation of ketamine specific complications that were found in this patient. Our case is unique given it highlights multiple complications of ketamine abuse, only one of which is completely understood.
